# Long-Term Administration of LL-37 Can Induce Irreversible Rosacea-like Lesion

**DOI:** 10.3390/cimb45040177

**Published:** 2023-03-24

**Authors:** Chuanxi Zhang, Yumeng Kang, Ziyan Zhang, Heliang Liu, Hong Xu, Wenchen Cai, Xuemin Gao, Jie Yang

**Affiliations:** 1 Clinical Medical College, North China University of Science and Technology, Tangshan 063210, China; 2 School of Public Health, North China University of Science and Technology, Tangshan 063210, China

**Keywords:** rosacea, LL-37, inflammation, skin fibrosis, lesion, model

## Abstract

Rosacea is a chronic inflammatory skin disease whose late manifestations have not yet been clearly reported in animal models. The objective of this study is to describe the skin lesions and major histopathological changes in a rosacea-like phenotype in mice induced by prolonged LL-37 administration and furthermore, to assess the potential of long-term LL-37 administration in inducing irreversible rosacea-like skin lesion models. Balb/c mice were continuously injected intradermally with LL-37 every 12 h to induce a rosacea-like phenotype. After LL-37 injections were administered for 20 consecutive days, the area of rosacea-like lesions gradually expanded in the first 13 days, then entered a stable phase. Haematoxylin and eosin (H&E) and Van Gieson’s staining showed a high degree of inflammatory cell aggregation, thickening of the epidermis and dermis, and collagen deposition in large quantities. The results of immunofluorescence staining and Western blotting showed that the expression of α-SMA, TNF-α, vimentin, and COL1 in the skin of mice was significantly upregulated. Short-term LL-37 administration induced rosacea-like lesions that only featured the aggregation of inflammatory factors and thickening of the epidermis, whereas no collagen hyperplasia was observed, and a full recovery was noticed. However, rosacea-like skin lesions induced by long-term LL-37 administration did not completely recover. Our study compares rosacea-like lesions induced by short-term versus long-term LL-37 administration, and the results suggest that irreversible rosacea-like lesions can be induced by long-term LL-37 administration.

## 1. Introduction

Rosacea is a chronic inflammatory skin disease that occurs on the cheeks, nose, chin, forehead, and eyes and is characterized by recurrent episodes of flushing, persistent erythema, papules, pustules, telangiectasias, rosacea nodules, and phymatous changes [1]. Rosacea is morphologically divided into erythematotelangiectatic rosacea, papulopustular rosacea, phymatous rosacea, and ocular rosacea [2]. Erythematotelangiectatic rosacea, characterized by persistent erythema with intermittent flushing of the nose and cheeks, is usually the first symptom of rosacea. As the disease progresses, patients develop papules and pustules in their lesions and progress to papulopustular rosacea. If the patient is not treated in time, rosacea will develop into phymatous rosacea, and irreversible skin fibrosis may occur in the skin lesions on the nose, cheeks, chin and eyebrows, resulting in thickening of facial skin, mainly manifested as the occurrence of rhinophyma. This will greatly affect the patient’s life [3]. The incidence of rosacea exceeds 5% of the population worldwide [4]. Among affected populations, Caucasian people have the highest prevalence rate of 2–22%, as their skin is relatively more sensitive to sunlight [4,5,6]. In epidemiological studies, the incidence of rosacea in non-Caucasian people residing in Asia, Africa, and South America has been found to have gradually increased to 10%, and such people of color often have late detection due to their darker skin color [7]. As a result, the optimal window for treatment is often missed, resulting in serious damage to the middle of the face accompanied by psychological distress [8].

Antimicrobial peptides (AMP) are natural compounds produced by prokaryotic and eukaryotic cells, which feature a wide range of antimicrobial activities targeting bacteria, viruses, fungi, and protozoa [9]. LL-37 is one such member of the AMP family that has been successfully isolated from a variety of species, including plants, insects, amphibians, fish, and mammals [10]. In addition, LL-37 is the only known human cathelicidin AMP (CAMP) produced by the cleavage of human cationic antimicrobial peptide-18 (HCAP-18) by kallikrein 5 and is comprised of 37 amino acids derived from the C-terminus of HCAP-18 α, resulting in the compound having a molecular weight of 4.5 kDa [11,12,13]. LL-37 exerts its antimicrobial activity through chemotaxis, the inhibition of neutrophil apoptosis, the stimulation of angiogenesis, tissue regeneration, and cytokine release [14]. Moreno Angatita et al. reported that LL-37 and its precursor hCAP18 are expressed in a variety of cell types, including epithelial cells and myeloid cells, in response to tissue damage, UV exposure, and microbial infections [15]. In normal skin tissue, LL-37 is mainly produced by keratinocytes, such that when bacteria, viruses, and other microorganisms invade the skin tissue, LL-37 is produced to destroy the outer walls of bacteria and viruses by binding to them, thereby killing invading microorganisms to exert its antimicrobial effect [16]. LL-37 can also play an anti-inflammatory role by blocking the binding of lipopolysaccharides (LPS) to TLR4, thereby inhibiting the activation of either the downstream NF-κB signaling pathway or MAPK signaling pathway to reduce the production of inflammatory factors [17]. Interestingly, previous studies have found that high concentrations of LL-37 promote the production of pro-inflammatory factors such as IL-1β and TNF-α to exacerbate inflammation, while large amounts of LL-37 have also been measured in the skin of patients with inflammatory skin diseases such as rosacea; in contrast, its presence is undetectable in healthy people [18]. Yamasaki’s study found that LL-37 was highly expressed in rosacea-like mouse models, and hence, the compound is considered a key molecule in the pathogenesis of rosacea [19]. A recent study found that the intradermal injection of LL-37 (300 μM) into mice could induce the infiltration of inflammatory cells in an NLRP3-dependent manner, thereby participating in the pathogenesis of rosacea [20].

Yamasaki and his team developed LL-37-induced rosacea-like mouse models in 2007. These established models included a KLK-5-induced inflammation model, a croton oil inflammation model, a 12-O-Tetradecanoylphorbol-13-acetate inflammation model, an arachidonic acid inflammation model, an RTX-induced vasodilation model, and a UVB-induced model, all of which represented the pathophysiology of rosacea to some extent [21]. In addition, Xue Luo et al. utilized demodex mites to induce rosacea-like lesions in rabbits [22]. However, the above rosacea-like models mainly manifested short-term inflammatory responses that were reversible. This has led to the current absence of animal models of papulopustular rosacea (PPR) and phymatous rosacea (PHR) [23].

Current research on rosacea is mainly based on an intradermal LL-37 injection-induced rosacea mouse model, which involves induced inflammation. Existing studies have constructed this model by the intradermal injection of LL-37 every 12 h for two consecutive days [19,24,25,26]. In this study, the feasibility of using long-term LL-37 administration to establish an irreversible mouse rosacea-like skin lesion model was explored by comparing the pathophysiological changes caused by its short-term versus long-term administration.

## 2. Materials and Methods

### 2.1. Animal Experiments

Twenty-three 4-week-old BALB/c males were supplied courtesy of Vital River Laboratory Animal Technology Co., Ltd. (Beijing, China). Adaptive feeding was carried out for 2 weeks before experimentation. LL-37 (24,461; Cayman Chemical Company, Ann Arbor, MI, USA) was administered intradermally to establish a rosacea-like mouse model. All animal experiments were approved by the Institutional Animal Protection and Use Committee of North China University of Science and Technology (LX2019033) and met the guidelines set by the Guide for the Care and Use of Laboratory Animals by the National Institutes of Health.

Six-week-old BALB/c mice weighing approximately 20 g each were shaved of their back hair the day prior to the experiment. Mice were then randomly divided into 3 groups: the long-term LL-37 administration group, the short-term LL-37 administration and self-recovery group, and the long-term LL-37 administration and self-recovery group. In the long-term LL-37 administration group, 15 mice received 40 μL of LL-37 [27] injected intradermally every 12 h for 20 days, and the changes in the back lesions of mice on days 0, 3, 5, 8, 10, 13, 15, 18, and 20 were recorded; 5 mice were then euthanized on days 0, 3, and 20. In the short-term LL-37 administration and self-recovery group, 15 mice were injected intradermally with 40 μL of LL-37 every 12 h for 2 days and then observed without intervention for 2 more days; the mice were photographed, and the changes in their back skin lesions were recorded on days 0, 1, 2, 3 and 4. Then, 5 mice were euthanized on days 0, 2, and 4. In the long-term LL-37 administration and self-recovery group, 15 mice were intradermally injected with 40 μL of LL-37 every 12 h for 10 days and then observed without intervention for another 10 days; the mice were photographed, and changes in their back skin lesions were recorded on days 0, 3, 5, 8, 10, 13, 15, 18 and 20. Then, 5 mice were sacrificed on days 0, 10, and 20. After the mice were anesthetized and euthanized, their back skins were excised and fixed with 4% paraformaldehyde before being paraffin-embedded. The paraffin sections were made to be 5 μm thick.

### 2.2. Histological Examination of Skin Tissue

The prepared paraffin sections (see Section 2.1) were stained with hematoxylin and eosin (H&E staining; Staining BA4025, BaSO Diagnostics Inc., Zhuhai, China). The H&E-stained sections were then observed for skin tissue morphology under a 200× microscope, and the total epidermis thickness was measured using the software ImageJ. Van Gieson staining (VG staining; BA4084, BaSO Diagnostics Inc., Zhuhai, China) in conjunction with the software ImageJ was used to analyze changes in the area of collagen deposition according to the manufacturer’s instructions.

### 2.3. Immunofluorescence Staining

The skin tissue sections were dewaxed and hydrated. After antigen retrieval treatment, anti-α-SMA antibodies (1:100 dilution, ab5694, Cambridge, UK), anti-TNF-α antibodies (1:100 dilution, GTX110520, San Antonio, TX, USA), or anti-vimentin antibodies (1:200 dilution, ab92547, Cambridge, UK) were added dropwise to the tissues and incubated overnight at 4 °C. Sheep anti-murine antibody (H + L) FITC (121,051, SeraCare, Milford, MA, USA) was then added at 37 °C for 1 h. Then, the tissues were stained with a dilution of 5 mg/mL 4′,6-diamidino-2′-phenylindole (DAPI; 14,285, Cayman Chemical Company, Ann Arbor, MI, USA) for 5 min. An Olympus DP80 microscope (Olympus, Hamburg, Germany) was used to observe the expression sites and levels of α-SMA, TNF-α, and vimentin. The obtained images were analyzed using cellSens Imaging Software v.1.8 (Olympus, Hamburg, Germany). Green fluorescence was taken to indicate α-SMA/TNF-α/vimentin expression.

### 2.4. Western Blot

Large proteins were isolated from mouse cells using a radioimmunoprecipitation assay (RIPA) buffer. Western blotting was then carried out. The main primary antibodies included TNF-α (1:1000 dilution, GTX110520; GeneTex, San Antonio, TX, USA), vimentin (1:4000 dilution, ab92547, Abcam, Cambridge, UK), α-SMA (ab5694; Abcam, Cambridge, UK), collagen 1 (COL1, 1:4000 dilution, ab34710, Abcam, Cambridge, UK), and β-actin (AC026; ABclonal, Wuhan, China). They were then incubated with goat anti-rabbit or anti-mouse secondary antibodies (074–1506/074-1806, Kirkegaard and Perry Laboratories, Gaithersburg, MD, USA). Immunoblot target bands were visualized using ECL Prime Western Blotting Detection Reagent (ZD310A, ZomanBio, Beijing, China). β-Actin was used as an internal reference.

### 2.5. Statistical Analysis

Statistical analyses were performed using SPSS 20.0 software (IBM Corp., Armonk, NY, USA) and GraphPad 8.0 software. Two-group comparisons were made using the unpaired Student’s *t*-test, while multiple-group comparisons were made using a one-way analysis of variance (ANOVA) followed by Tukey’s post hoc test. Statistical significance was defined as *p* < 0.05 with a 95% confidence interval.

## 3. Results

### 3.1. The Degree of Rosacea-like Skin Lesions Induced by Long-Term LL-37 Administration Was More Severe Than Those Induced by Short-Term Administration

**Figure 1:**
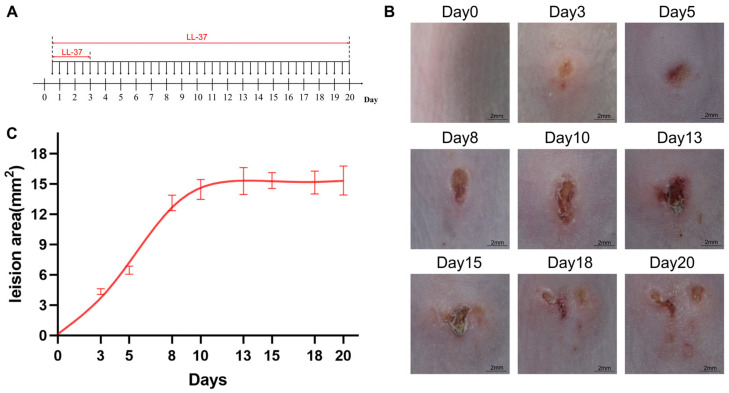
(**A**) The long-term LL-37 administration group received intradermal injections of LL-37 every 12 h before being euthanized on days 0, 3, and 20. (**B**) Mouse lesion area and erythema changes over time induced by LL-37 intradermal injections on days 0, 3, 5, 8, 10, 13, 15, 18, and 20 (scale bar = 2 mm). (**C**) Trend plot of lesion area. With the increase in the number of LL-37 injections, the area of erythema first expanded and then stabilized. Data are presented as the mean ± SD; *n* = 5 for each group.

**Figure 2:**
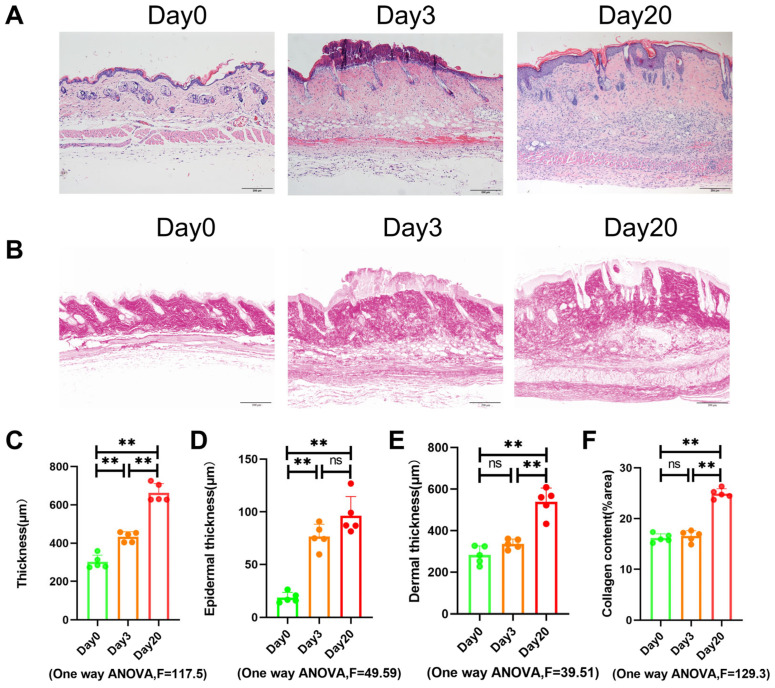
LL-37-induced rosacea-like lesions presented with epidermal thickening without collagen density changes in the early stage and dermal thickening with increased collagen density in the late stage. (**A**,**C**–**E**) Hematoxylin-eosin (H&E) staining of lesioned skin (scale bar = 200 μm). (**B**,**F**) Representative skin sections stained with Van Gieson staining (VG staining) (scale bar = 200 μm). Data are presented as the mean ± SD; *n* = 5 for each group. ** *p* < 0.01.

**Figure 3:**
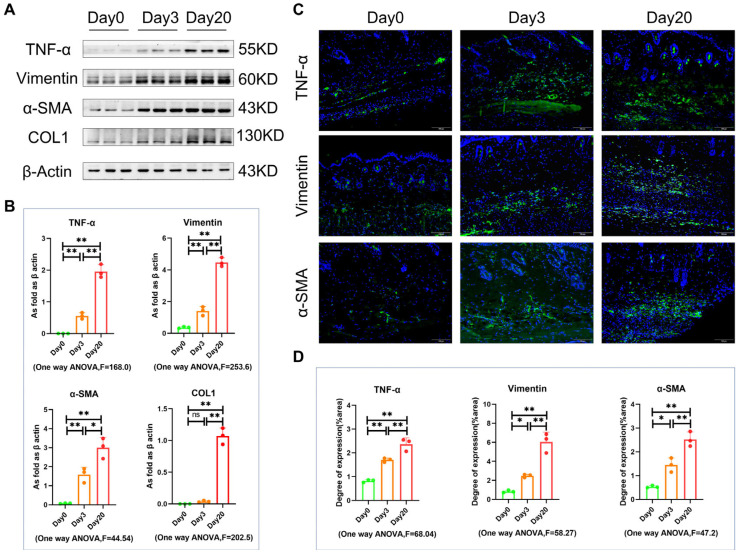
(**A**,**B**) Western blotting detected the relative protein expression levels of TNF-α, vimentin, α-SMA, and COL1 in the skin of mice on days 0, 3, and 20 of LL-37 administration. (**C**,**D**) Immunofluorescence method to detect the relative expression levels of TNF-α, vimentin, and α-SMA in mouse skin. Data are presented as the mean ± SD; *n* = 3 for each group. * *p* < 0.05, ** *p* < 0.01.

In order to observe whether the long-term administration of LL-37 could induce the late fibrosis phenotype of rosacea in mice, we administered intradermal injections of LL-37 over 20 days (Figure 1A). We first recorded changes in the areas of small back lesions on days 0, 3, 5, 8, 10, 13, 15, 18, and 20 during the administration of the intradermal LL-37 injections (Figure 1B). From this, we found that LL-37 induced apparent rosacea symptoms, erythema, and telangiectasia on the skin. The average erythema area gradually expanded with the increasing number of administered LL-37 injections over 0–10 days. The erythema area then stabilized after 13 days with the continued administration of LL-37 injections (Figure 1C). In addition, the skin thickness and collagen density under long-term administration on days 0, 3, and 20 were compared by H&E staining and VG staining (Figure 2A,B). The results showed that compared to day 0, LL-37 increased the total skin thickness on the 3rd day due to the thickening of the epidermis, though the dermis did not change significantly. On the 20th day, both the epidermis and dermis were significantly thickened; the thickness of the epidermis was not significantly different from day 3, while that of the dermis was (Figure 2C–E). The results of VG staining showed no significant change in collagen density on day 3, in contrast to its increase on day 20 (Figure 2F). These results indicate that the rosacea-like skin of model mice induced by LL-37 administration was mainly due to epidermal thickening in the early stage and dermal thickening in the late stage. This thickening may be caused by late collagen deposition. Tumor necrosis factor α (TNF-α) is an inflammatory cytokine produced by macrophages/monocytes during inflammation [28]. Vimentin is an important component of the cytoskeleton that is specifically present in fibroblasts in the skin [29]. Smooth muscle alpha-actin (α-SMA) is a hallmark of myofibroblasts. In our mouse rosacea model, TNF-α, vimentin, and α-SMA were all significantly upregulated on days 3 and 20, and their expression levels on day 20 were much higher than on day 3; in contrast, the expression of type I collagen was not significantly upregulated on day 3, while it was upregulated on day 20 (Figure 3A,B). In addition, the immunofluorescence results indicated the upregulation of TNF-α, vimentin, and α-SMA expression, which validated the immunoprotein blotting results, and these factors were mainly expressed in the dermis layer (Figure 3C,D). These findings reveal that with the prolongation of LL-37 administration, the infiltration of inflammatory cells and the release of inflammatory factors in the mouse dermis increased. The early upregulation of α-SMA and vimentin may be caused by the proliferation of capillaries, and the increase in type I collagen appeared on the 20th day, indicating that the long-term LL-37-induced model of rosacea may involve myofibroblast proliferation and fibrosis.

### 3.2. Rosacea-like Lesions Induced by Short-Term LL-37 Administration Were Able to Recover Spontaneously

**Figure 4:**
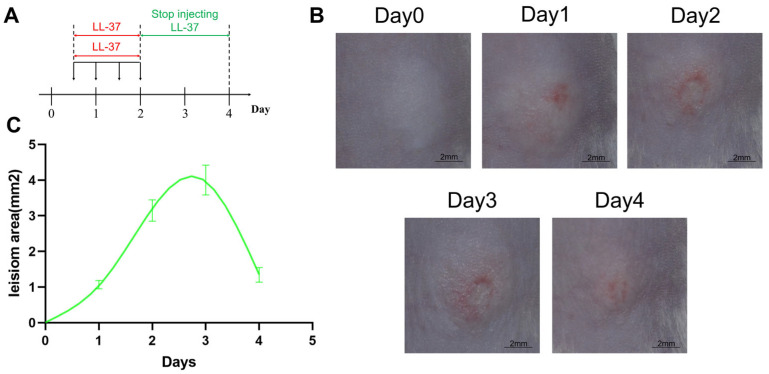
(**A**) The short-term LL-37 administration and self-recovery group received intradermal injections of LL-37 every 12 h for 2 days before being euthanized on days 0, 2, and 4. (**B**) LL-37 intradermal injections were administered for 2 days and then stopped for 2 days, during which erythema changes in the lesion areas of mice on days 0, 1, 2, 3, and 4 from the first injection were recorded (scale bar = 2 mm). (**C**) Trend plot of lesion area. With the extension of LL-37 injection time, the erythema area first expanded; after stopping the injection, the erythema area continued to expand and then gradually shrunk, and there was still a trend of recovery up until the 4th day. Data are presented as the mean ± SD; *n* = 5 for each group.

**Figure 5:**
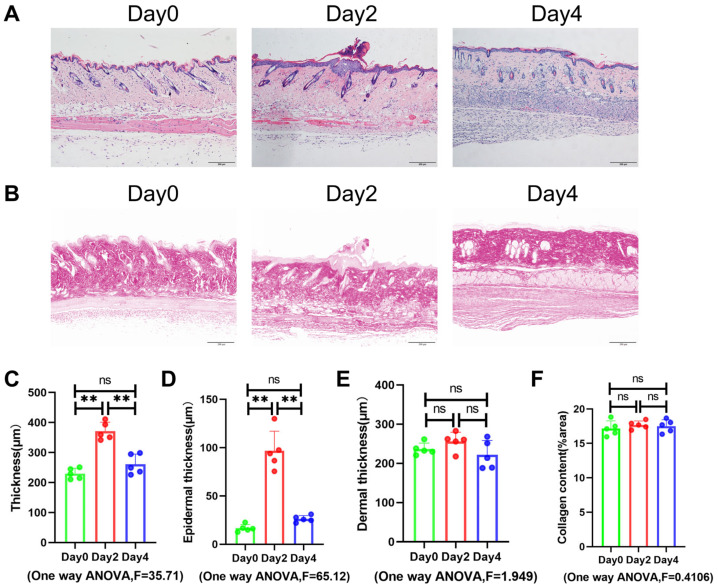
The rosacea-like skin lesions induced by LL-37 short-term administration mainly involved epidermal thickening without collagen hyperplasia, and the epidermis essentially returned to normal level after 2 days without LL-37 administration. (**A**,**C**–**E**) Hematoxylin-eosin (H&E) staining of lesioned skin (scale bar = 200 μm). (**B**,**F**) Representative skin sections stained with Van Gieson staining (VG staining) (scale bar = 200 μm). Data are presented as the mean ± SD; *n* = 5 for each group. ** *p* < 0.01.

**Figure 6:**
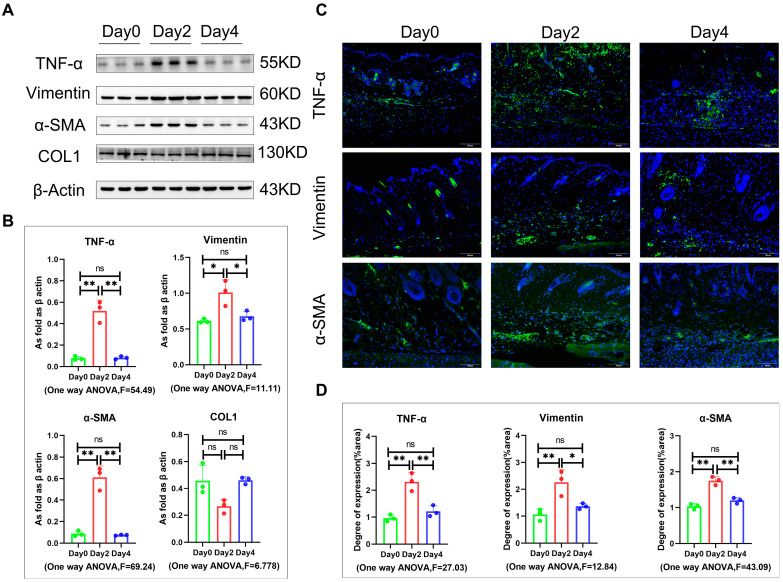
(**A**,**B**) Western blotting detected the expression of TNF-α, vimentin, α-SMA, and COL1 in the skin of mice on days 0, 2, and 4. (**C**,**D**) Immunofluorescence method to detect the immunofluorescence expression of TNF-α, vimentin, and α-SMA in mouse skin. Data are presented as the mean ± SD, *n* = 3 for each group. * *p* < 0.05, ** *p* < 0.01.

In order to investigate whether the rosacea-like lesions induced by short-term LL-37 administration could recover spontaneously, we first induced the mature rosacea-like model by intradermal LL-37 injections for two consecutive days [18] and then observed the lesions without any intervention for two more days (Figure 4A). We found that the erythema area gradually expanded under the administration of LL-37 for the first two days, then gradually decreased after stopping the injection of LL-37 on day 3. The trend of erythema area reduction on day 4 was still significant (Figure 4B,C), indicating the possibility of complete recovery of rosacea-like skin lesions induced by short-term LL-37 administration. By comparing the results of H&E staining of the mouse lesion skin on days 0, 2, and 4 (Figure 5A), it was found that the skin thickening induced by short-term LL-37 administration mainly occurred in the epidermis, which largely returned to normal thickness after stopping LL-37 administration (Figure 5C–E). In addition, the VG staining results of skin lesions on days 0, 2, and 4 showed no significant change in collagen density (Figure 5B,F), indicating that there was no obvious collagen deposition in the rosacea-like skin lesions induced by short-term LL-37 administration. The Western blotting and immunofluorescence staining results showed that TNF-α, vimentin, and α-SMA were mainly expressed in the dermis, exhibiting upregulated expression levels on day 2, and returned to baseline on day 4 (Figure 6). There was no significant difference in the expression level of type I collagen on days 0, 2, and 4 (Figure 6A,B). The above results indicate that the rosacea-like skin lesions induced by short-term LL-37 administration involved the aggregation of inflammatory cells, the release of inflammatory factors, telangiectasia, and hyperplasia but could return to normal levels after the cessation of LL-37 administration.

### 3.3. Rosacea-like Lesions Induced by Long-Term LL-37 Administration Did Not Fully Recover on Their Own

**Figure 7:**
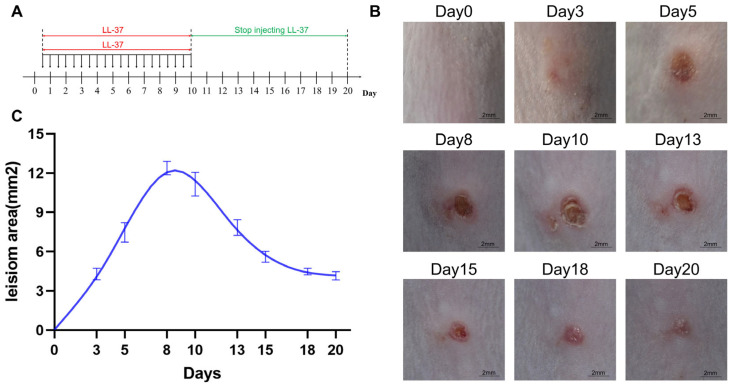
(**A**) The long-term LL-37 administration and self-recovery group received intradermal injections of LL-37 every 12 h for 10 days before being euthanized on days 0, 10, and 20. (**B**) LL-37 intradermal injections were stopped after 10 days, and erythema changes in the lesion areas of mice on days 0, 3, 5, 8, 10, 13, 15, 18, and 20 from the first injection were recorded (scale bar = 2 mm). (**C**) Trend plot of lesion area. With the extension of LL-37 injection time, the erythema area gradually expanded, and after stopping the injections, the erythema area stabilized after returning to a certain level on the 18th day but did not fully recover. Data are presented as the mean ± SD; *n* = 5 for each group.

**Figure 8:**
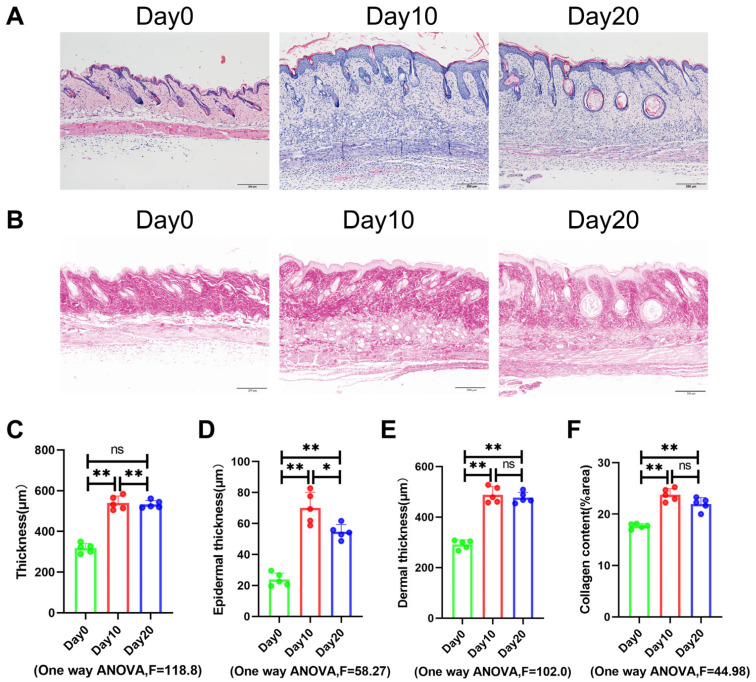
Rosacea-like skin lesions induced by 10 days of LL-37 administration exhibited both epidermis and dermis thickening accompanied by an increase in collagen density, and neither the epidermis nor the dermis could return to normal thickness after halting administration for 10 days. (**A**,**C**–**E**) Hematoxylin-eosin (H&E) staining of lesioned skin (scale bar = 200 μm). (**B**,**F**) Representative skin sections stained with Van Gieson staining (VG staining) (scale bar = 200 μm). Data are presented as the mean ± SD; *n* = 5 for each group. * *p* < 0.05, ** *p* < 0.01.

**Figure 9:**
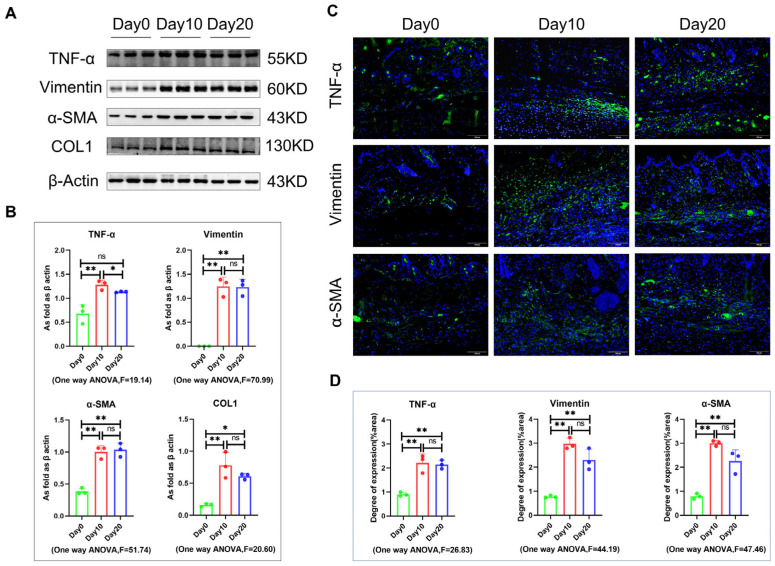
(**A**,**B**) Western blotting detected the relative protein expression levels of TNF-α, vimentin, α-SMA, and COL1 in the skin of mice on days 0, 10, and 20. (**C**,**D**) Immunofluorescence method to detect the relative expression levels of TNF-α, vimentin, and α-SMA in mouse skin. Data are presented as the mean ± SD; *n* = 3 for each group. * *p* < 0.05, ** *p* < 0.01.

To observe whether prolonged LL-37 administration could develop irreversible rosacea-like lesions, we first induced the lesions by administering LL-37 intradermal injections for 10 consecutive days, which was followed by 10 days without intervention (Figure 7A). We found that as the LL-37 injection time increased, the erythema area gradually increased in tandem, and after stopping the injections, the erythema area gradually recovered, but its recovery rate slowed down over time. The erythema area stabilized after returning to a certain level on day 18 and then failed to fully recover (Figure 7B,C). This suggests that the long-term administration of LL-37 may result in persistent rosacea-like lesions. By comparing the H&E staining results of the skin lesions taken on days 0, 10, and 20 (Figure 8A), it was found that LL-37 continued to induce lesions for 10 days, and the epidermis and dermis of the skin from these lesion sites were significantly thickened. After halting administration for 10 days, the epidermis thickness was slightly reduced compared to the 10th day but did not return to baseline, while the dermis thickness did not change significantly when compared to the 10th day (Figure 8C–E). This indicates that the epidermis of rosacea-like lesions induced by LL-37 administration for 10 days has a certain reversible hyperplasia, while dermal hyperplasia cannot fully recover on its own. In addition, the VG staining results of the skin lesions on days 0, 10, and 20 showed that the collagen density increased significantly on the 10th day of LL-37 administration when compared to baseline, after which there was no further significant change in collagen density after 10 days of stopping administration (Figure 8B,F), indicating that the long-term administration of LL-37 not only induces collagen deposition but also that this effect cannot be completely autonomously reversed. The results of Western blotting and immunofluorescence staining showed that the expression levels of TNF-α, vimentin, and α-SMA were significantly upregulated on day 10 when compared to baseline, while there was no significant change on day 20 when compared to day 10 (Figure 9). The immunoblotting results showed that the expression of type I collagen was significantly upregulated on day 10 while remaining largely unchanged from day 10 until day 20 (Figure 9A,B). The above results suggest that rosacea-like lesions induced by long-term LL-37 administration may persistently cause the release and stimulation of inflammatory factors, fibroblast proliferation, telangiectasia and proliferation, and collagen deposition.

## 4. Discussion

Rosacea is a chronic skin condition of unknown etiology that initially progresses from erythema to papular pustules and then eventually to phymatous rosacea. Rosacea presents with erythema, papules, pustules, and telangiectasias in the early stages and can still be treated with topical brimonidine, azelaic acid, and lasers. If rosacea is not controlled in time, skin fibrosis may occur, causing irreversible thickening of the skin, which can only be treated by surgical excision. However, surgical removal usually leaves a certain facial scar. Therefore, this study induced irreversible rosacea-like lesions, which provides a basis for establishing animal models of phymatous rosacea in the future. In this study, we used LL-37 to induce rosacea-like lesions in the skin of mice. By observing the erythema area change curve, epidermis and dermis thickness, collagen density, expression levels of α-SMA, TNF-α, vimentin, and type I collagen of the groups of mice subjected to either the short-term or long-term LL-37 administration, it was found that lesions induced by short-term administration could essentially fully recover without further intervention. However, long-term administration caused not only inflammatory factor aggregation and capillary hyperplasia but also the thickening of both the epidermis and epidermis with collagen hyperplasia. Moreover, the above pathophysiological changes induced by long-term LL-37 administration were persistent and did not fully recover on their own.

In constructing the mouse models, we consulted previous studies that had established murine models of rosacea, in which LL-37 injections were injected every 12 h for two consecutive days to induce rosacea-like lesions in mice [19,30,31]. Therefore, in order to observe what would happen to rosacea-like skin lesions induced by long-term LL-37 administration, its duration of intradermal administration was adjusted to 20 days, while the remaining details of the injection methods and intervals were made to remain consistent with previous studies and finally compared to the model involving only 3 days of injection (short-term administration). The rosacea-like skin lesions induced by short-term LL-37 administration exhibited erythema and edema on the back skin of the mice, while H&E staining revealed thickening of the epidermis, a high degree of inflammatory cell infiltration and capillary hyperplasia in the subcutaneous tissue layer, and increased expression of TNF-α [26]. These findings were consistent with the appearance of the skin taken from lesion sites on day 3. However, the area of rosacea-like lesions induced by continuous administration over 20 days stabilized within a certain range after the 13th day and no longer enlarged thereafter, indicating that the development of rosacea-like lesions entered a plateau after 13 days. The morphology, Western blotting, and immunofluorescence results jointly showed that the rosacea-like skin lesions induced by long-term LL-37 administration exhibited simultaneous thickening of the epidermis and dermis with a high degree of collagen hyperplasia and deposition. This outcome differed from the presentation of rosacea-like lesions induced by short-term LL-37 administration [32] but was similar to what we have seen in previous studies employing intradermal injections of LL-37 every 24 h for 20 days [33]. In addition, in studies on bleomycin-induced skin fibrosis, collagen deposition was found to be accompanied by the thickening of both the dermis and epidermis, as well as the upregulation of α-smooth muscle actin and wave protein expression levels [34,35]. In our constructed mouse rosacea model, in addition to the inflammatory manifestations, the above manifestations of skin fibrosis also appeared, which indicates that the rosacea progressed from inflammation to skin fibrosis and further indicates that it is feasible to construct a model of advanced rosacea fibrosis by prolonging the duration and frequency of LL-37 intradermal injections.

Previous studies have found that the rosacea model induced by the short-term administration of LL-37 is characterized by extensive infiltration of inflammatory cells into the dermis and epidermal hyperplasia [36]. This is consistent with our findings. However, few previous studies have observed whether rosacea-like skin lesions induced by short-term LL-37 administration can recover completely on their own, while most studies have explored the therapeutic effect of a drug of interest on rosacea, such as Xin Yuan et al., who discussed the therapeutic effect of artemisinin on rosacea [32]. Therefore, after the successful induction of rosacea-like lesions by short-term LL-37 administration, we observed the lesions for 2 days without further intervention and compared them to short-term model mice. The epidermal hyperplasia, telangiectasia, and aggregation of inflammatory factors observed in the rosacea-like skin lesions induced by short-term LL-37 administration spontaneously recovered. This suggests that its short-term administration did not induce fibrosis. Unlike these short-term induced lesions, those induced by long-term LL-37 administration were not observed to significantly improve in terms of hyperplasia of the dermis and epidermis after 10 days of observation without intervention, and the accumulation of inflammatory factors, fibroblast proliferation, and collagen fiber deposition did not improve significantly. This indicates that the long-term administration of LL-37 to induce a rosacea model may cause irreversible damage and result in relatively stable fibrosis.

In summary, our study shows that the long-term administration of LL-37 can induce irreversible rosacea-like lesions. Rosacea-like skin lesions induced by short-term administration were mainly characterized by thickening of the epidermis and accumulation of inflammatory factors and could essentially recover on their own. In comparison, rosacea-like lesions induced by long-term LL-37 administration also displayed dermal thickening, collagen deposition, and fibroblast proliferation but could not recover on their own. Therefore, our study provides a novel method for establishing a more stable model of rosacea fibrosis and also provides a basis for studying advanced lesions in the treatment of rosacea.

## Data Availability

Not applicable.
